# Efficacy of spinal ultrasonography just before caudal epidural block for identifying tethered cord syndrome in urological cases with sacral dimples: a retrospective descriptive study

**DOI:** 10.1007/s00540-025-03478-x

**Published:** 2025-03-07

**Authors:** Fumio Watanabe, Taiki Kojima, Mitsunori Miyazu, Hiroshi Kitoh

**Affiliations:** 1 Department of Anesthesiology, Aichi Children’s Health and Medical Center, 426 Nana-Chome, Morioka-Cho, Obu, Aichi 478-8710 Japan; 2 Department of Comprehensive Pediatric Medicine, Nagoya University Graduate School of Medicine, Aichi, Japan

**Keywords:** Dimple, Caudal epidural block, Spinal ultrasound, Spinal MRI

## Abstract

**Purpose:**

Tethered cord syndrome (TCS) can be detected on spinal ultrasound (s-US) performed by anesthesiologists immediately prior to caudal epidural block. In such cases, neurosurgical consultation should be considered to ensure timely diagnosis and treatment. This study aimed to describe: (1) the frequency of TCS requiring surgery in pediatric urological cases with sacral dimples following neurosurgical consultation; (2) the sacral dimple morphology indicative of TCS; and (3) filum terminale thickness as a predictor of TCS.

**Methods:**

This retrospective, single-center, descriptive study included children ≤ 3 years old with sacral dimples undergoing their first urological surgery with caudal epidural block between April 2019 and June 2024. We described: (1) the proportion of cases requiring spinal surgery based on s-US and postoperative magnetic resonance imaging (MRI); (2) differences in the proportions of patients with dimple long diameter ≥ 5 mm and distance from anal margin ≥ 25 mm between TCS and non-TCS cases; and (3) the optimal filum terminale thickness for predicting TCS using receiver operating characteristic curve analysis.

**Results:**

Among 130 patients analyzed, 6 (4.6%) underwent tethered cord release surgery based on abnormal findings from s-US and postoperative MRI. A significant difference in the proportion of patients with dimple long diameter ≥ 5 mm was identified between TCS and non-TCS cases (*p* = 0.046). A cutoff filum terminale thickness ≥ 1.3 mm yielded a Youden index of 0.73, with 93% sensitivity and 80% specificity for detecting filum terminale lipoma.

**Conclusion:**

Spinal ultrasonography and dimple size may help to identify underlying TCS.

## Introduction

Tethered cord syndrome (TCS) can be detected during spinal ultrasound performed by anesthesiologists just before caudal epidural block [1]. If spinal ultrasound findings suggest TCS, consultation with a neurosurgeon should be considered to ensure the patient does not miss an opportunity for timely treatment. However, no studies have shown the results of consultations with neurosurgeons for TCS discovered during caudal epidural block. Avoiding puncture of a tethered spinal cord is crucial to performing safe caudal epidural block. TCS can be effectively identified by detecting a low-lying conus medullaris and increased thickness of the filum terminale within the sacrum. We, therefore, developed a method of systematic spinal ultrasound screening (s-US) for the lumbar and sacral spine that can be performed in 3–5 min [2].

Not all cases with dimples have undergone examinations to detect TCS prior to the planned surgeries. Moreover, the actual probability of TCS being present is relatively low, even among children with dimples [3]. Caudal epidural block is considered safe to perform on dimple cases, provided no TCS are present. However, no gold standard has yet been defined for the detection of TCS in cases where dimples were noted for the first time just prior to performing caudal epidural block.

Children with urological diseases show a relatively high incidence of TCS [4], which often represents a contraindication for caudal epidural block [5]. Caudal epidural block remains a widely accepted technique for providing analgesia during lower abdomen and perineal surgeries in urological cases. As a result, dimples that indicate TCS should be carefully assessed in urological cases before proceeding with caudal epidural block to avoid iatrogenic spinal cord injuries.

The morphology of the dimple and spinal ultrasound may be helpful in detecting TCS. The following cutoff values for dimple morphology have been used as potential risk factors for TCS, particularly among neonates: (a) dimple size ≥ 5 mm and (b) distance from the anal margin to the edge of the dimple (dimple-anus distance) ≥ 25 mm [6]. Among children over 6 months old with advanced ossification of the posterior vertebral arches, spinal magnetic resonance imaging (s-MRI) is preferred over spinal ultrasound. Conversely, the posterior walls of the sacrum are thin enough for ultrasound to penetrate, even in children over 6 months old [1]. This means that TCS extending to the sacral canal can be observed using spinal ultrasound through the sacrum and lumbar intervertebral spaces without the posterior arches in these children. A filum terminale thicker than 1.1 mm on ultrasound indicates the possibility of filum terminale lipoma [7]. This measurement criterion can thus be utilized in the sacrum to detect filum terminal lipoma, even in children over 6 months old.

We aimed to describe: (1) the frequency of TCS cases requiring surgical intervention, as detected by spinal ultrasound, in urological patients with sacral dimples; (2) the sacral dimple morphology indicative of TCS; and (3) the filum terminale thickness suggestive of TCS.

## Methods

### Study design and setting

This study was conducted and reported in accordance with the Strengthening the Reporting of Observational Studies in Epidemiology (STROBE) statement. This retrospective, single-center, descriptive study investigated the period from 1 April 2019 to 30 June 2024 at Aichi Children’s Health and Medical Center, a tertiary-care hospital in Japan. This study was approved by the institutional ethics committee (approval no. 2024023; date of approval: 1 July 2024). The requirement for written consent was waived by the institutional ethics committee based on the retrospective nature of this study.

### Inclusion and exclusion criteria

This study included patients ≤ 3 years old with dimples identified immediately prior to caudal epidural block, who were scheduled to undergo their first urological surgeries during the study period. In the absence of preoperative s-MRI, s-US was performed just before the caudal epidural block to evaluate for underlying TCS. Enrolled patients were categorized into two groups: (1) Group T, in which spinal abnormalities potentially causing TCS were detected on postoperative s-MRI; and (2) Group N, which included cases where no spinal abnormalities were identified on s-US or where abnormalities were suspected on s-US but not confirmed on postoperative MRI. The indications for caudal epidural block included urethroplasty for hypospadias, orchidopexy for cryptorchidism, ureterocystoneostomy for vesicoureteral reflux, renal pelvis reconstruction surgery for hydronephrosis, and endoscopic urethrotomy for urethral stricture.

The exclusion criteria were as follows: (1) patients who had previously undergone spinal surgeries before first urological surgery; (2) patients who had not undergone postoperative s-MRI when s-US detected spinal abnormalities.

### Systematic spinal ultrasound screening (convenient conus to coccyx point-of-care ultrasound)

**Fig. 1:**
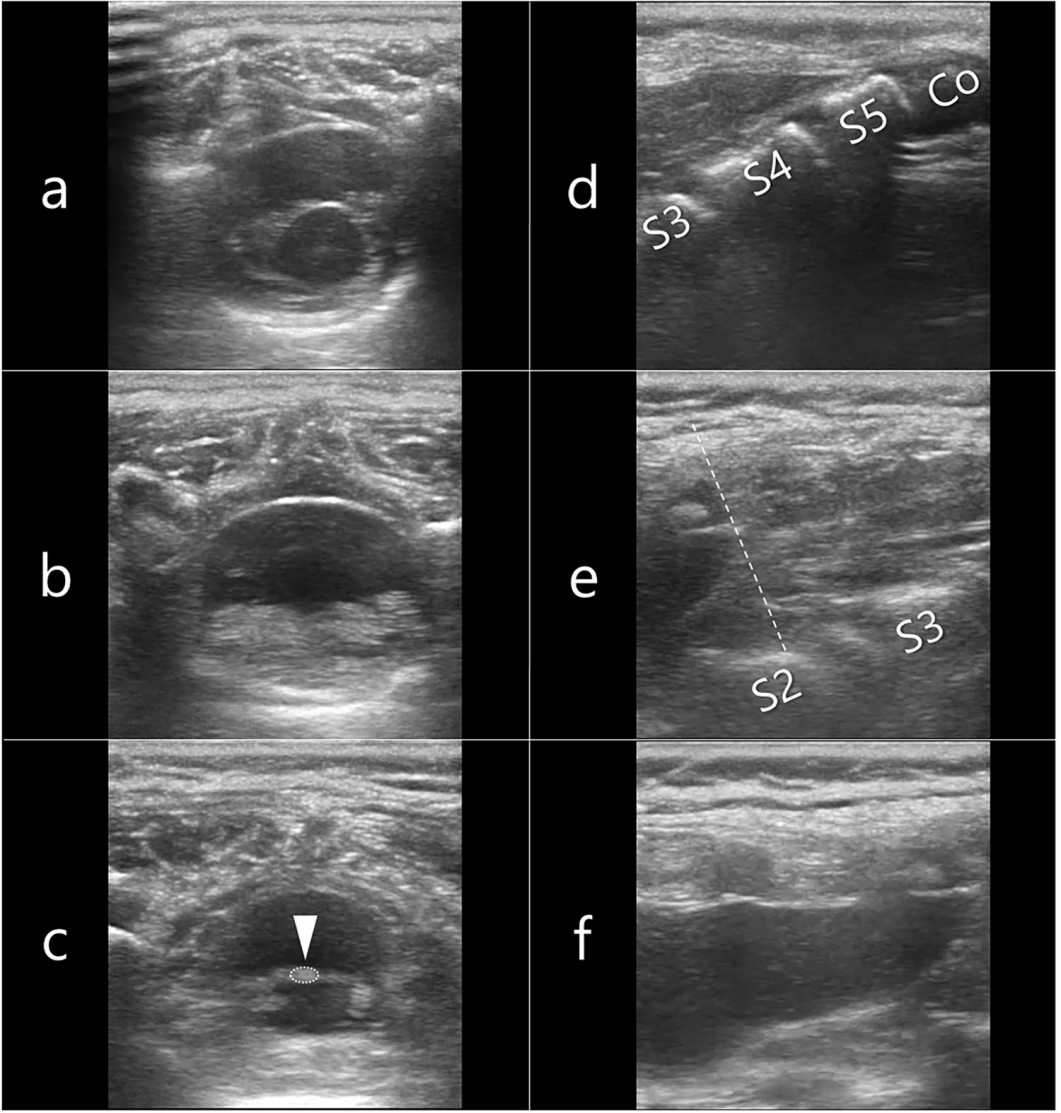
Key imaging findings from systematic spinal ultrasound screenings of a 16-month-old child. **a** Horizontal image between T12 and L1, showing the conus medullaris and cauda equina. **b** Horizontal image between L2 and L3, predominantly displaying the cauda equina within the dural sac, as the conus medullaris typically terminates at the L2 level. **c** Horizontal image between L5 and S1, where the filum terminale (arrow) can be identified due to dispersion of the cauda equina. **d** Sagittal image on which the sacral level can be determined by the position of the coccyx. **e** Sagittal image on which the structure in the sacral canal can be assessed, and the end of the dural sac (dotted line) can be identified. **f** Sagittal image showing structures within the dural sac, including the filum terminale. *Co* coccyx, *S* sacrum

At our institution, s-US serves as the standard procedure for detecting TCS during the evaluation of patients with dimples. We performed s-US using the previously reported method [2]. Even if ossification of the vertebral posterior arch is present, structures within the spinal canal and sacral canal can be observed using spinal ultrasound through the lumbar intervertebral spaces and sacrum as shown in Fig. 1. We conducted caudal epidural block in cases where no TCS were detected. All s-US was performed with the patient in a lateral decubitus position after completing the induction of general anesthesia. First, utilizing the lowest rib (the 12th thoracic vertebra: T12) as a reference point, we systematically acquired continuous horizontal ultrasound images of the spinal cord extending from T12 to the coccyx using a sliding ultrasound probe. Second, we assessed the sacral canal in the sagittal plane. The current method can determine the following: (1) the lowest point of the conus medullaris (usually situated cranial to L2 in normal anatomy); (2) the lowest point of the dural sac (typically cranial to S2 in normal anatomy); (3) the width of the filum terminale, using a value < 1.1 mm for differentiation from filum terminale lipoma; and (4) the presence of cysts and lipomas in the sacral canal. We utilized HSL25x^®^ ultrasonography kits (6–13 MHz, linear; FUJIFILM Sonosite, Bothell, WA, USA). The quality of spinal ultrasonography was ensured by having trained pediatric anesthesiologists perform the procedure. A senior anesthesiologist received training in s-US from pediatric radiologists. Pediatric anesthesiologists who had undergone more than five on-the-job training sessions with the senior anesthesiologist performed the spinal ultrasound. In addition, the senior anesthesiologist reviewed all spinal ultrasound images either during the procedure or on the same day the procedure was performed.

### Spinal magnetic resonance imaging

For postoperative s-MRI, a 1.5-T system (Ingenia; Philips Medical Systems, Best, the Netherlands) was used, with T1- and T2-weighted images in both short- and long-axis views employed to diagnose TCS. Consultation with a neurosurgeon was conducted by pediatric urologists to assess the risk of future neurogenic bladder development based on patient characteristics in cases where TCS was suspected from s-US findings and dimple morphology. The neurosurgeons performed postoperative s-MRI, referencing the s-US images shared in the electronic medical records. Imaging parameters for T1-weighted s-MRI were as follows: repetition time, 475 ms; echo time, 11 ms; flip angle, 90°; section thickness, 5 mm; matrix size, 288 × 169; and field of view, 180 mm. The imaging parameters for T2-weighted s-MRI were: repetition time, 4,439 ms; echo time, 120 ms; flip angle, 90°; section thickness, 5 mm; matrix size, 288 × 104; and field of view, 180 mm. Neurosurgeons reviewed the MRI findings and diagnosed filum terminale lipoma if the filum terminale showed signal hyperintensity on axial T1-weighted images.

### Data collection

Demographic data recorded from electronic medical records included age, sex, height, body weight, diagnosed urological diseases, gestational age, and birth weight. We assessed the risk of potential TCS by measuring the size of the dimple and its distance from the anus as part of standard care, and we recorded this information in the anesthesia records for communication with neurosurgeons and urologists. Dimple size and dimple-anus distance were measured after positioning the patient in the left lateral decubitus position for caudal epidural block, using a flexible paper ruler that conforms to the curvature of the gluteal region. Information regarding dimples was collected from anesthesia records, including: (1) short and long diameters of the dimple; and (2) dimple-anus distance. The dimple was predominantly elongated along the cephalocaudal axis, with the longitudinal extent along this axis constituting the long diameter, and the transverse extent (from left to right) constituting the short diameter.

### Collection and analysis of data derived from spinal ultrasound screening and spinal magnetic resonance imaging

In this study, we recorded key anatomical landmarks, including the lowest points of the conus medullaris and dural sac, as well as measurement of the filum terminale obtained from both s-US and s-MRI. In addition, we recorded the specific spinal diseases diagnosed from s-US and s-MRI. A filum terminale thickness ≥ 1.1 mm was considered as filum terminale thickening [7]. The minimum filum terminale thickness was defined as 0.1 mm, corresponding to the limit of detection for the ultrasound device employed. We tried to describe the optimal cutoff value for filum terminale thickness measured by s-US in detecting filum terminale lipoma, as conclusively diagnosed by s-MRI.

### Adverse events related to caudal epidural block

Ropivacaine was administered for caudal epidural block at a maximum dose of 3 mg/kg, diluted to concentrations ranging from 0.25 to 0.1875%. Adverse events of caudal epidural block such as local anesthetic toxicity, cerebrospinal fluid aspiration, lower limb motor impairment, and sensory disturbances, were extracted from medical and anesthesia charts.

### Proportion of cases in which spinal surgery was performed

We described the proportion of cases in which spinal surgery for TCS was performed based on the results of s-US and s-MRI.

### Sacral dimple morphology

We described the number of cases in Groups T and N with: (a) long diameter of the dimple ≥ 5 mm; and (b) dimple–anus distance ≥ 25 mm [6].

### Filum terminale thickness

We described the number of cases in Groups T and N with filum terminale thickness ≥ 1.1 mm [7]. In addition, we tried to determine the optimal cutoff value for filum terminale thickness as measured by s-US for detecting filum terminale lipoma.

### Statistical analysis

The patient demographics and clinical characteristics were compared between Groups T and N. The outcome measures are presented as median with interquartile range (IQR) or as count and percentage. The Mann–Whitney U-test was utilized to assess continuous variables with skewed distributions, while the χ^2^ test or Fisher’s exact test was used for comparing categorical variables to assess differences in patient characteristics between groups. The following cutoff values were assessed using the *χ*^2^ test: (a) long diameter of the dimple ≥ 5 mm; and (b) dimple-anus distance ≥ 25 mm. We used the receiver operating characteristic (ROC) curve to determine the optimal cutoff value for filum terminale thickness measured by s-US in detecting filum terminale lipoma, as conclusively diagnosed by s-MRI. Youden’s index is used to identify the value that offers maximal sensitivity and specificity on the ROC curve [8]. We calculated Youden’s index to determine the optimal cut-off for detecting spinal abnormalities from s-US. Patients with missing values were excluded. Data were analyzed utilizing STATA 17.0 (StataCorp, College Station, TX, USA), with a two-sided *p* value < 0.05 indicating statistical significance.

## Results

### Proportion of cases in which spinal surgery was performed

**Fig. 2:**
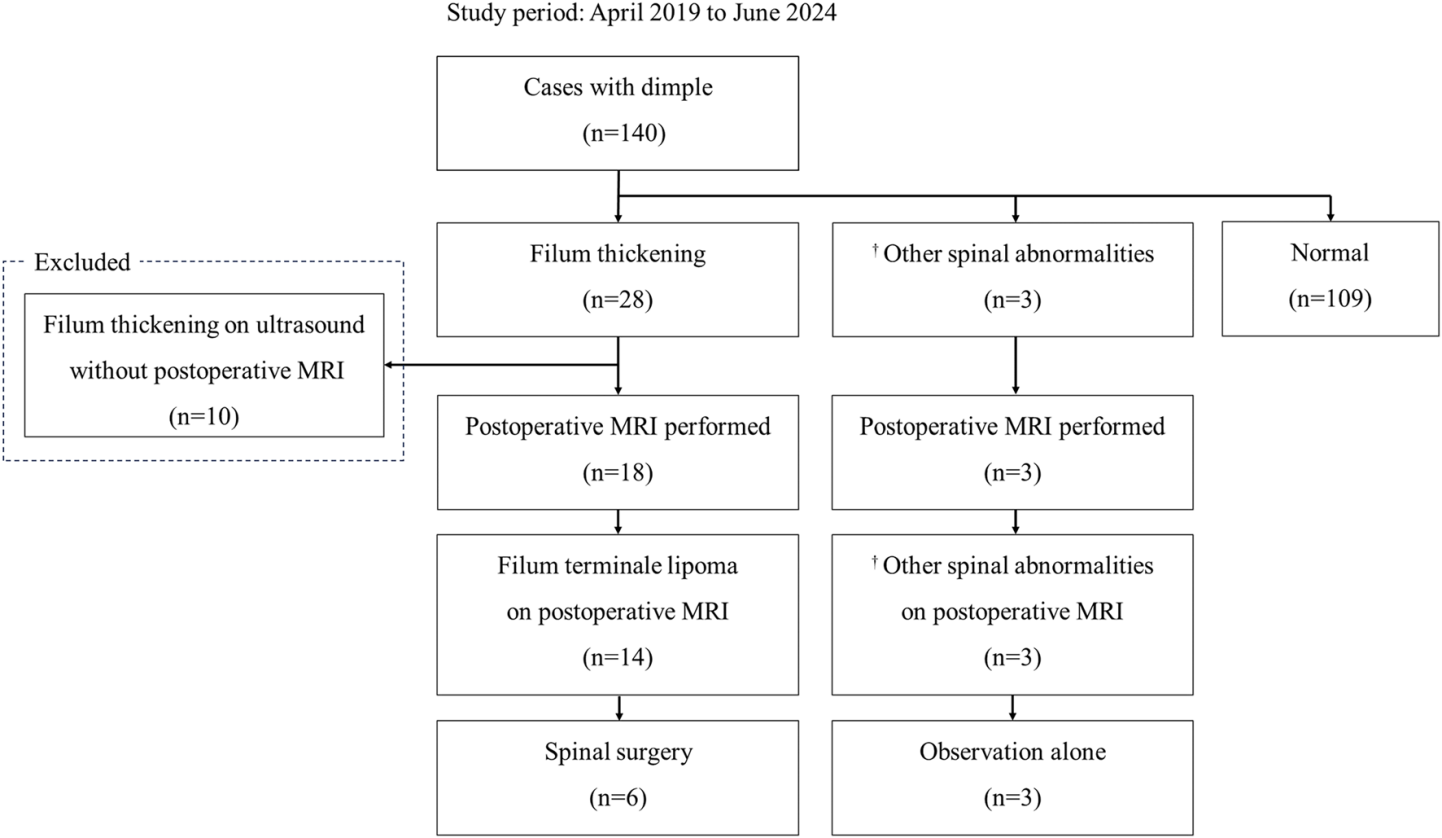
Flow of enrolled patients and diagnosis on spinal ultrasound and spinal MRI. †Other spinal abnormalities include two cases with spinal cysts and one case with a low-lying dural sac continuous with sacral dermal sinus

**Fig. 3:**
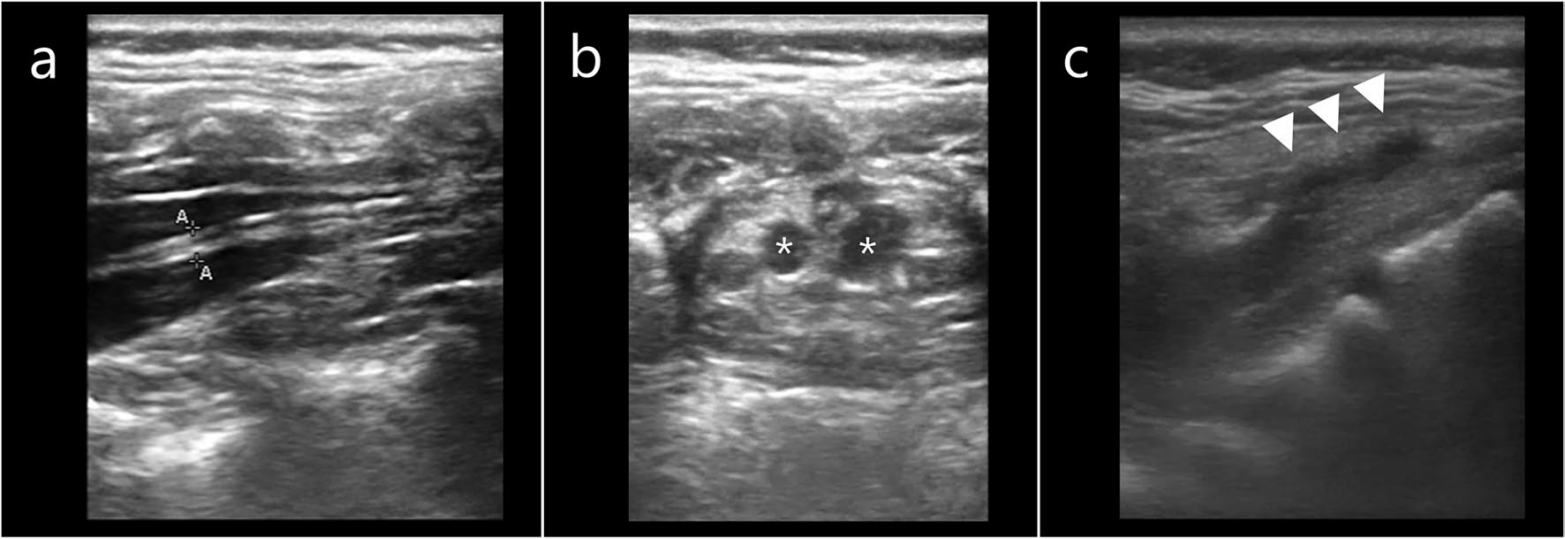
Spinal abnormalities identified on spinal ultrasound. **a** Spinal ultrasound of a 22-month-old child with filum terminale lipoma. The filum terminale is 2.0 mm thick and surrounded by hyperechoic fatty tissue. **b** Spinal ultrasound of a 12-month-old child showing Tarlov cysts (*). **c** Spinal ultrasound showing a low-lying dural sac at the S4 level, continuous with a dermal sinus (arrows)

A total of 140 cases of dimples were identified during the period from April 2019 to June 2024 as shown in Fig. 2. Using s-US, 28 cases were diagnosed with filum terminale thickening, two cases with spinal cysts, one case with a low-lying dural sac continuous with sacrococcygeal dermal sinus. Figure 3 shows the ultrasound images of spinal abnormalities identified on s-US. Among the 28 cases with filum thickening identified on s-US, 18 cases underwent postoperative s-MRI. The 10 cases with filum terminale thickening identified by s-US but without postoperative s-MRI were excluded, resulting in the analysis of 130 cases. Among the excluded patients were those for whom the attending urologist considered neurosurgical consultation unnecessary due to the absence of confirmed neurogenic bladder. Among 18 cases for which postoperative s-MRI was performed due to filum terminale thickening by s-US, 14 cases were diagnosed with filum terminale lipoma, and 6 cases (4.6% of 130 cases with dimples) were scheduled for tethered cord release surgery. Among the six cases that underwent spinal cord untethering surgery, five showed a low-lying conus medullaris at L3, and the remaining had a low-lying dural sac. Two cases of spinal cysts and one case of a low-lying dural sac continuous with a sacrococcygeal dermal sinus, initially detected by s-US, were confirmed postoperatively using s-MRI. Patient characteristics for the 130 cases are presented in Table 1. The 17 patients in Group T and 113 patients in Group N showed no significant differences in background characteristics.

**Table 1:** Patient characteristics (*N* = 130)

	Group T^†^ (*n* = 17)	Group N^‡^ (*n* = 113)	*p* value
Patient characteristics			
Age, months	14 (11–22)	17 (12–23)	0.51
Sex, male	16 (94.1)	108 (95.6)	0.79
Body weight, kg	9.4 (8.0–10.1)	9.8 (8.6–11.2)	0.28
Gestational age group			
Pre-term	5 (29.4)	33 (29.2)	0.94
Term	10 (58.2)	63 (55.8)	
Post-term	2 (11.8)	17 (15.0)	
Primary diagnosis			
Hypospadias			
Proximal type	7 (41.2)	38 (33.6)	0.46
Distal type	4 (23.5)	34 (30.9)	
Vesicoureteral reflux	1 (5.9)	3 (2.7)	
Cryptorchidism	4 (23.5)	29 (25.7)	
Hydronephrosis	0 (0.0)	8 (7.1)	
Urethral stricture	1 (5.9)	1 (0.9)	

Data are presented as median (IQR) or number (percentage)

^†^Group T, spinal abnormalities suspected as representing tethered cord syndrome on postoperative MRI

^‡^Group N, normal without spinal abnormalities

*IQR* interquartile range, *MRI* magnetic resonance imaging

One case in Group N showed a low-lying conus medullaris at L3, but that patient was diagnosed as a normal variant due to the absence of filum terminale thickening. Patients with filum terminale thickness ≥ 1.1 mm were significantly more frequent in Group T than in Group N, as shown in Table 2.

**Table 2:** Sacrococcygeal abnormality and width of filum terminale (*N* = 130)

	Group T^†^ (*n* = 17)	Group N^‡^ (*n* = 113)	*p* value
Low-lying conus medullaris	5 (29.4)	1 (0.9)	< 0.001
Width of filum terminale^§^			
≥ 1.1 mm	14 (82.4)	10 (8.9)	< 0.001
< 1.1 mm	3 (17.7)	103 (91.2)	
Long diameter of the dimple			
≥ 5 mm	17 (100.0)	91 (80.5)	0.046
< 5 mm	0 (0.0)	22 (19.5)	
Dimple-anal distance			
≥ 25 mm	8 (47.1)	66 (58.4)	0.38
< 25 mm	9 (52.9)	47 (41.6)	
Short diameter of dimple, mm	4 (3–5)	3 (3–4)	0.20

Data are presented as median (IQR) or number (percentage)

^†^Group T, spinal abnormalities suspected as representing tethered cord syndrome on postoperative MRI

^‡^Group N, normal without spinal abnormalities

^§^Width of filum terminale: Group T included 3 cases without filum terminale thickening (2 cases with spinal cysts, 1 case with a low-lying dural sac)

*IQR* interquartile range

### Association between dimple morphology and tethered cord syndrome

The χ^2^ test revealed a significant difference in the proportion of patients with a long diameter of the dimple ≥ 5 mm between Groups T and N, whereas no significant difference in the proportion of patients with a dimple–anus distance ≥ 25 mm was found (Table 2). Nine cases in which spinal abnormalities were detected showed a dimple-anal distance < 25 mm.

### Optimal cutoff for filum terminale thickness in s-MRI

**Fig. 4:**
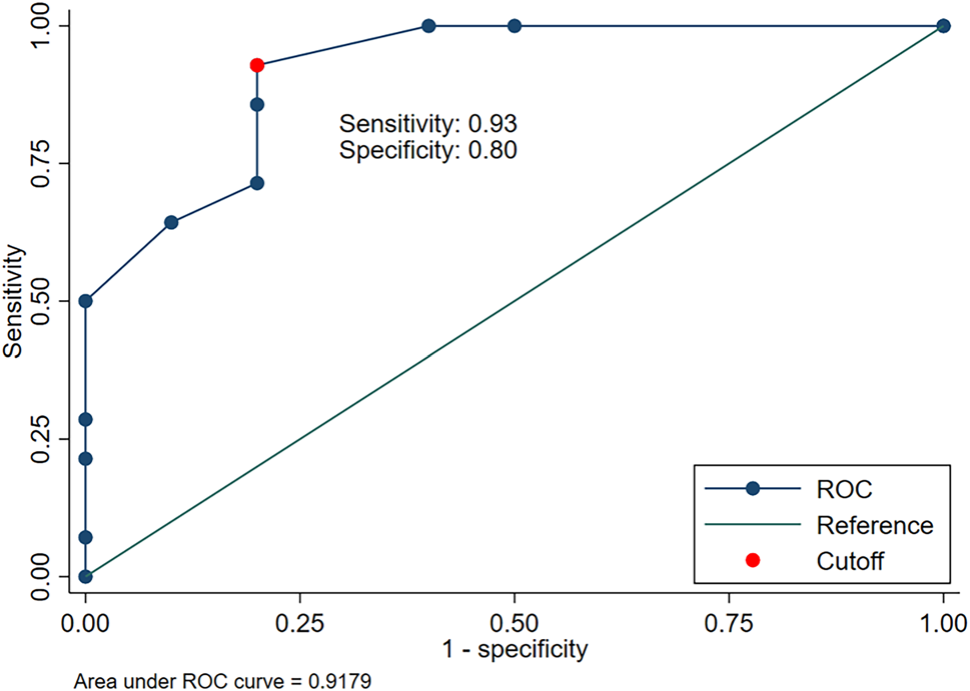
ROC curve demonstrating the relationship between filum terminale lipoma detected on MRI and filum terminale thickness as measured by spinal ultrasound. *ROC* receiver operating curve

The ROC curve demonstrated the relationship between filum terminale lipoma detected on s-MRI and filum terminale thickness as measured by s-US as shown in Fig. 4. A cutoff thickness of 1.3 mm resulted in the maximum Youden index of 0.73, with 93% sensitivity and 80% specificity for detecting filum terminale lipoma. The area under the curve (AUC) was 0.92 (95% confidence interval, 0.81–1.00).

### Adverse events related to caudal epidural block

None of the enrolled cases experienced any of the following adverse events: local anesthetic toxicity, cerebrospinal fluid aspiration, lower limb motor impairment, or sensory disturbance.

## Discussion

Urological patients in whom a sacral dimple is identified prior to performing caudal epidural block have the potential for underlying TCS and may require surgical intervention. To avoid missed diagnostic opportunities, s-US is recommended in such cases. This is an effective modality for diagnosing TCS, even in children over 6 months old. A sacral dimple with a long-axis diameter ≥ 5 mm may be indicative of spinal abnormalities associated with TCS.

In this study, s-US was able to detect spinal abnormalities even in children > 6 months old with advanced ossification of the vertebral posterior arches. The posterior walls of the sacrum were still thin enough for ultrasound to penetrate into the sacral canal, even in those patients. Spinal ultrasound thus appears primarily applicable to infants < 4–6 months old without the ossification of the posterior arch. MRI then becomes the preferred modality for children > 6 months old, in whom ossification limits complete evaluation of the spine via ultrasound. Certainly, s-US may miss localized spinal abnormalities under the ossified posterior arches. However, s-US could catch TCS, which extends from the conus medullaris to the sacrococcygeal region, by observing the tethered cord from the intervertebral space and sacrum. In this study, the frequency of TCS requiring surgical intervention was 4.6% in urological cases with dimples. This value is higher than previously reported (0.7–1.4%) from studies examining all newborns with cutaneous abnormalities, including dimples [3, 9, 10]. When examining children in outpatient settings who may cry and have difficulty remaining still, performing detailed assessments of the sacrococcygeal region and locating dimples can be challenging. In addition, performing s-US on restless children requires a high level of expertise. On the other hand, detailed examination of the sacrococcygeal region and performance of s-US in a short amount of time are obviously easier under general anesthesia. Missing the diagnosis of TCS in postoperative patients with hypospadias can lead to the worst-case scenario of neurogenic bladder, requiring intermittent catheterization. Performing s-US for dimples found just before caudal epidural block may help avoid overlooking TCS and lead to the necessary consultations with neurosurgeons.

The classical dimple morphologies suggested to be associated with a high risk of spinal abnormalities are dimple length ≥ 5 mm and dimple-anal distance ≥ 25 mm [6]. In this study, long diameter of a dimple contributed to predicting TCS. Furthermore, this study showed the optimal cutoff value of a filum terminale width as measured by s-US was 1.3 mm to distinguish filum terminal lipoma in patients ≤ 3 years old. The classical cutoff of 2 mm for filum terminale thickening on spinal ultrasound in neonates has been criticized for insufficient sensitivity, with some suggesting a cutoff of 1.1 mm [7]. The cutoff value of 1.1 mm is applicable even to infants over 6 months, although the filum terminale thickens due to the gradual accumulation of fat [11]. However, patients in this study had a median age of 16 months and the appropriate cutoff for filum terminale thickness was 1.3 mm. Further investigation is needed regarding the cutoff values suitable for different target populations.

Previous studies have reported that caudal epidural block for hypospadias resulted in an increased frequency of postoperative complications compared to dorsal penile block [12]. However, the application of caudal epidural block for hypospadias remains controversial due to unadjusted confounding factors and differences in patient severity and surgical procedures between subgroups in those studies [13]. A randomized controlled trial showed no significant difference in postoperative complications between caudal epidural block and dorsal penile nerve blocks [14]. Caudal epidural block is, therefore, expected to continue to see wide use in urological surgeries, and enhancing the safety of this procedure will be essential. Such enhancement would include the detection of TCS contradicting the use of this technique.

This retrospective, single-center, descriptive study showed several important limitations. First, the results cannot be generalized because data were collected from a single institution. Second, this study exhibited selection bias because postoperative s-MRI was not performed in some cases where s-US suggested thickening of the filum terminale. As a result, the frequency of potential TCS may have been higher than suggested in the results. Third, reporting bias may have been present in the measurement of dimple morphology and thickness of the filum terminale due to the self-reported nature of data collection by attending anesthesiologists. However, the assessment of the risk of TCS was conducted within limited clinical timeframes and the measured values are representative of real clinical practice. Fourth, due to the limited sample size of this study, we did not assess the calibration of our prediction tool. The area under the ROC curve was used solely to illustrate the relationship between filum terminale lipoma detected on s-MRI and filum terminale thickness**.** Calibration represents an important consideration for assessing the reliability of predictions, so this limitation should be addressed in future research. In this study, the number of cases in which spinal MRI was performed was very limited. To demonstrate the utility of spinal ultrasound, a prospective study needs to be conducted in which both spinal ultrasound and spinal MRI are performed in all cases showing a sacral dimple, to compare findings between modalities.

In conclusion, for children with sacral dimples, s-US under general anesthesia prior to caudal block may help to identify TCS. This approach may enhance the diagnostic accuracy and clinical management of sacral dimples. However, further prospective studies are needed to validate the efficacy of the present findings. To ensure generalizability, large-scale, comprehensive studies stratified by age, sex, and ethnicity will be required.

## Data Availability

A data availability statement is not applicable, as this study does not involve a dataset that can be shared.

## References

[1] Eizaga Rebollar R, Carnota Martín AI, Borreiros Rodríguez E, Rojo DR. Tethered spinal cord syndrome discovered during ultrasound-guided caudal block. Anesthesiology. 2024;140(6):1203–4. 38498665 10.1097/ALN.0000000000004899

[2] Watanabe F, Miyazu M, Kojima T. Introduction of convenient conus to coccyx point-of-care ultrasound (C3PO) in children with a sacral dimple. J Clin Anesth. 2021;74: 110446. 34225186 10.1016/j.jclinane.2021.110446

[3] Ausili E, Maresca G, Massimi L, Morgante L, Romagnoli C, Rendeli C. Occult spinal dysraphisms in newborns with skin markers: role of ultrasonography and magnetic resonance imaging. Childs Nerv Syst. 2018;34(2):285–91. 29075839 10.1007/s00381-017-3638-0

[4] Martínez-Frías ML. Spina bifida and hypospadias: a non-random association or an X-linked recessive condition? Am J Med Genet. 1994;52(1):5–8. 7977461 10.1002/ajmg.1320520103

[5] Wiegele M, Marhofer P, Lönnqvist PA. Caudal epidural blocks in paediatric patients: a review and practical considerations. Br J Anaesth. 2019;122(4):509–17. 10.1016/j.bja.2018.11.030. (**Epub 2019 Feb 1. PMID: 30857607; PMCID: PMC6435837**). 30857607 10.1016/j.bja.2018.11.030 PMC6435837

[6] Kriss VM, Desai NS. Occult spinal dysraphism in neonates: assessment of high-risk cutaneous stigmata on sonography. AJR Am J Roentgenol. 1998;171(6):1687–92. 9843314 10.2214/ajr.171.6.9843314

[7] Shin HJ, Kim MJ, Lee HS, Kim HG, Lee MJ. Optimal filum terminale thickness cutoff value on sonography for lipoma screening in young children. J Ultrasound Med. 2015;34(11):1943–9. 10.7863/ultra.14.10079. (**Epub 2015 Sep 18 PMID: 26384611**). 26384611 10.7863/ultra.14.10079

[8] Hua J, Tian L. A comprehensive and comparative review of optimal cut-points selection methods for diseases with multiple ordinal stages. J Biopharm Stat. 2020;30(1):46–68. 10.1080/10543406.2019.1632876. (**Epub 2019 Jun 28 PMID: 31250693**). 31250693 10.1080/10543406.2019.1632876

[9] Chern JJ, Kirkman JL, Shannon CN, Tubbs RS, Stone JD, Royal SA, Oakes WJ, Rozzelle CJ, Wellons JC. Use of lumbar ultrasonography to detect occult spinal dysraphism. J Neurosurg Pediatr. 2012;9(3):274–9. 22380955 10.3171/2011.12.PEDS11351

[10] Kucera JN, Coley I, O’Hara S, Kosnik EJ, Coley BD. The simple sacral dimple: diagnostic yield of ultrasound in neonates. Pediatr Radiol. 2015;45(2):211–6. 24996813 10.1007/s00247-014-3110-1

[11] Albakheet SS, Yoon H, Lee MJ, Kim MJ, Park EK, Shim KW, Kim DS, Eun HS, Han K, Shin HJ. Determining the optimal timing of screening spinal cord ultrasonography to detect filum terminale lipoma in infants. Ultrasonography. 2020;39(4):367–75. 32962332 10.14366/usg.19061 PMC7515663

[12] Kundra P, Yuvaraj K, Agrawal K, Krishnappa S, Kumar LT. Surgical outcome in children undergoing hypospadias repair under caudal epidural vs penile block. Paediatr Anaesth. 2012;22(7):707–12. 21957982 10.1111/j.1460-9592.2011.03702.x

[13] Anand S, Braga LH. Fragility index of meta-analyses and randomized-controlled trials on the efficacy of caudal block for hypospadias: is the glass half-full or half-empty? J Pediatr Urol. 2023;19(3):359–60. 36907750 10.1016/j.jpurol.2023.02.005

[14] Alizadeh F, Amraei M, Haghdani S, Honarmand A. The effect of caudal epidural block on the surgical complications of hypospadias repair in children aged 6 to 35 months: a randomized controlled trial. J Pediatr Urol. 2022;18(1):59.e1-6. 34887183 10.1016/j.jpurol.2021.11.009

